# Impact of household income on the risk of overweight and obesity over time among preschool-aged children: a population-based cohort study

**DOI:** 10.1186/s12889-024-18010-1

**Published:** 2024-02-21

**Authors:** Yi-Chang Chou, Feng-Shiang Cheng, Shih-Han Weng, Yung-Feng Yen, Hsiao-Yun Hu

**Affiliations:** 1 Department of Education and Research, Taipei City Hospital, No.145, Zhengzhou Rd., Datong Dist., 103212 Taipei, Taiwan; 2 University of Taipei, Taipei, Taiwan; 3 Institute of Public Health, National Yang Ming Chiao Tung University, Taipei, Taiwan; 4 Section of Infectious Diseases, Taipei City Hospital, Yangming Branch, Taipei, Taiwan; 5 Department of Health Care Management, National Taipei University of Nursing and Health Sciences, Taipei, Taiwan

**Keywords:** Low-income household, Overweight/obesity, Preschool-aged children

## Abstract

**Background:**

The temporality of household income level with overweight/obesity in children has not been extensively studied. Little research has been conducted to determine the impact of household income on the risk of childhood overweight/obesity over time. This population-based cohort study aimed to investigate the impact of household income on the risk of overweight/obesity over time among preschool-aged children in Taiwan.

**Methods:**

From 2009 to 2018, we recruited 1,482 preschool-aged children ( ≦ 7 y of age) from low-income households and selected age- and sex-matched controls from non-low-income households for comparison; All participants were selected from those who consistently participated in the Taipei Child Development Screening Program and were monitored for overweight/obesity using body mass index (BMI) until December 31, 2018. Low-income households were defined as those with an average monthly disposable income < 60% of the minimum standard of living expense in Taiwan. The primary outcome was childhood overweight or obesity in study participants, defined as BMI (kg/m^2^) ≥ 85th percentile or ≥ 95th percentile, respectively. The generalized estimating equations (GEE) model was used to determine the impact of low-income households on the risk of overweight/obesity in study participants.

**Results:**

Over 21,450 person-years of follow-up, 1,782 participants developed overweight /obesity, including 452 (30.5%) and 1,330 (22.4%) children from low- and non-low-income households, respectively. The GEE model showed that the first group had a significantly higher risk of becoming overweight/obese than the other during the follow-up period (adjusted odds ratio [aOR] = 1.44, 95% CI: 1.29–1.60). Moreover, children of foreign mothers had a higher risk of becoming overweight/obese than those of Taiwanese mothers during the follow-up period (aOR = 1.51, 95% CI: 1.24–1.8). The subgroup analysis revealed a significant association between low-income households and an increased risk of overweight/obesity in children aged 2–7 years (*P* =.01). However, this association was not observed in children aged 0–1 years (*P* >.999).

**Conclusions:**

During the follow-up period, there was a notable correlation between low-income households and an increased risk of preschool-aged children developing overweight or obesity. Implementing health promotion initiatives aimed at reducing overweight and obesity in this demographic is crucial.

## Background

The association between socio-economic status (SES) and obesity is complex, varies by population groups and could change over time [1, 2]. Children from low SES were less likely to have shared family mealtimes, an important determinant to promote healthy behaviors and prevent overweight or obesity of children [3]. Children who are overweight or obese are at a high risk of remaining overweight or obese into adulthood, which could increase the risk of chronic comorbidities and mortality [4–7].

Some studies have shown that household income is negatively correlated with body weight of children [8–14]. However, most of the evidence regarding the relation of household income with childhood obesity came from cross-sectional studies [8, 9, 13]. A previous report showed that there may be a bidirectional association between socio-economic status and obesity [15]. Therefore, a cohort study is important to determine the causality between socio-economic status and the risk of obesity.

Although poverty is an important social determinant of children’s health [16, 17], few longitudinal studies have determined the temporality of household income level with the risk of childhood obesity. Two previous longitudinal studies found that recurrent household poverty or children born into poverty had a 1.5–1.68 times higher risk of overweight/obesity than those who never experienced poverty during follow-up or those not born into poverty [10, 11]. Another cohort study found that poverty prior to age 2 years was associated with risk of obesity by age 15.5 years [12]. A recent prospective study from the USA has shown a similar gradient in obesity risk by household income [18]. However, previous studies measured participants’ household income level through self-report, which was not confirmed by the government official household income document [10–12]. Moreover, as the BMI is dynamic change over time in children, limited research has been conducted to determine the impact of household income on the risk of childhood overweight/obesity over time.

Understanding the impact of household income on the risk of childhood overweight/obesity would aid in devising future health promotion programs. However, there remains a dearth in studies on the relationship between household income and childhood obesity in Taiwan. Existing studies are cross-sectional studies [19–21] or studies focused solely on maternal information [22]. Therefore, we conducted a population-based longitudinal cohort study to evaluate the association of household income level with the risk of overweight/obesity over time in a large sample of preschool-aged children in Taipei, Taiwan.

## Methods

### Data source

This cohort study used Taipei Child Development Screening Program dataset and comprised pre-school age children who participated in the annual health checkups between 2009 and 2018 in Taipei. To evaluate child development, Taipei City Government provides free annual health checkups for children under the age of 7. Parents take their children to the hospital for the health checkup according to the established schedule. During the health checkup, the nurses collected baseline information on participants’ body mass index and sociodemographic factors.

This study accessed the Taipei Child Development Screening Program dataset in June 2023. The Taipei City Hospital Research Ethics Committee (no. TCHIRB- 11,205,011-E) approved the study protocol, and the requirement for informed consent was waived. All related procedures were performed in accordance with the relevant national and institutional guidelines and along with those stipulated in the Declaration of Helsinki.

### Study population

A total of 161,419 individuals participated in the health checkups between 2009 and 2018. To determine the impact of household income on the risk of overweight and obesity over the time, children included in this study were required to have at least two BMI measurements. Children belonging to low-income households, as determined by the Taipei City Government, were classified as low household income in this study, and the rest were classified as non-low-income households. Of these participants, 124,670 individuals underwent repeated examinations. After excluding individuals with congenital disease (*n* = 1,660) and those with incomplete covariate data (*n* = 5,130), 117,880 participants were recruited in the analysis, including 1,482 individuals from low-income household.

The subjects included in the control group in this study were selected from 117,880 study participants and matched for age and sex. Four controls were randomly selected for each individual from low-income household [23]. Control participants were excluded if they belonged to a low-income household. Finally, a total of 7,410 participants were included in this cohort. All participants from low-income and non-low-income households underwent follow up surveys for overweight/obesity until December 31, 2018.

### Main explanatory variable

The main explanatory variable was low-income households, which are defined as those with a monthly average per-member gross income of less than the monthly minimum living expense standard of Taiwan [≤ 19,013 New Taiwan Dollars (NTD)] [24]. Furthermore, the family property in low-income households in Taiwan must not exceed a certain amount (average movable assets (including savings, stocks, and investments) < NTD 150,000; real Estate < NTD 7,650,000), as determined by the central or municipal authorities in the corresponding year [24]. The qualifications for low income household are reviewed annually by the Taipei City Government. It has been recognized by government agencies and is more reliable than income information obtained from self-completed questionnaires.

### Outcome variable

The primary outcome was child overweight or obesity in study participants, defined as body mass index (BMI, kg/m^2^) ≥ 85th percentile(overweight) or ≥ 95th percentile (obesity), adjusted for age and sex, by the Growth Charts for Taiwanese Children [25]. BMI was calculated as weight in kilograms divided by height in meters squared.

### Covariates

The covariates in this study included age (0–1 years: 0–24 months; 2–7 years: 25–84 months), gender, mother’s nationality, dmft index of child (number of decayed teeth, missing due to caries and filled/restored due to dental caries for primary teeth [26]), and whether eye disorders were present and required prescription glasses. The mother’s nationality was classified as either Taiwanese or foreign. The dmft index of the child is an abbreviation for the number of decayed teeth that are missing due to caries and filled/restored due to dental caries for primary teeth. The dmft index was converted from records of oral examinations conducted by specialist physicians and ranged from 0 to 20. Vision examination results are categorized as either requiring spectacle wear or not.

### Statistical analysis

Baseline characteristics among participants with different level of household income were compared using Mann-Whitney U test for continuous variables and the Chi-square test for categorical variables, as appropriate.

Generalized estimating equations (GEE) were conducted to determine the impact of household income on the risk of overweight/obesity over the time after adjusting variables such as age, sex, mother’s nationality, baseline dmft index, and spectacle wear. The variable with *p* < 0.05 was defined as a significant factor associated with overweight/obesity in the multivariate analysis. Adjusted odds ratios (aOR) with 95% confidence intervals (CI) were reported to show the strength and direction of these associations.

We used Wilcoxon rank sum test to evaluate the variations in BMI with age in children from low-income households and non-low-income households. To examine the robustness of the main findings, subgroup analyses were conducted after stratifying study participants by age, gender, mother’s nationality, baseline dmft index, and spectacle wear. All data management and analyses were performed using the SAS 9.4 software package (SAS Institute, Cary, NC).

## Results

### Characteristics of the study population

This cohort study included 7,410 preschool-aged children in the analysis. The mean (standard deviation [SD]) number of repeated BMI measurements was 3.6 (1.5) times, with a mean follow-up duration for overweight/obesity of 2.9 (1.7) years. For all participants, the overall mean (SD) age were 3.0 (1.6) years, and 51.8% were male.

Table 1 shows the characteristics of the study population according to household income level. Compared to participants from middle- and high-income household, those from low-income household had a higher proportion of individuals with foreign mothers and wearing spectacles. Moreover, children from low-income household were more likely to be overweight/obese both at the first and last Child Development Screening Examination.

**Table 1:** Characteristics of the study population by the household income level (*N* = 7410)

Characteristics	Total, *n* (%)	Low-income household, *n* (%)			P-value			
		Yes (*n* = 1482)		No (*n* = 5928)				
Age, yrs, mean (SD)	3.03 (1.59)	3.03 (1.59)		3.03 (1.59)	> 0.999			
Male gender	3840 (51.8)	768 (51.8)		072 (51.8)	> 0.999			
Mother’s nationality								
Taiwanese	7116 (96.0)	1335 (90.1)		5781 (97.5)	< 0.001			
Foreigners	294 (4.0)	147 (9.9)		147 (2.5)				
Baseline BMI, Kg/m^2^, mean (SD)	16.21 (1.82)	16.48 (2.06)		16.15 (1.75) 1.88 (3.01)	< 0.001			
Baseline dmft index^a^, mean (SD)	2.13 (3.27)	3.12 (3.98)			< 0.001			
Spectacle wear	467 (6.3)	112 (7.6)		355 (6.0)	0.026			
Overweight/Obesity on the first examination	1702 (23.0)	430 (29.0)		1272 (21.5)	< 0.001			
Overweight/Obesity on the last examination	1782 (24.1)	452 (30.5)		1330 (22.4)	< 0.001			
Follow-up years, mean (SD)	2.85 (1.70)	2.83 (1.63)		2.86 (1.71)	0.745			

^a^dmft index: number of decayed teeth, missing due to caries and filled/restored due to dental caries for primary teeth

### Factors associated with overweight/obesity among children

After controlling for participants’ age, gender, mother’s nationality, baseline dmft index, and spectacle usage, GEE model showed that children from low-income households had a significantly higher risk of becoming overweight/obesity than those from middle- and high-income households during the follow-up period (aOR: 1.44, 95% CI: 1.29–1.60) (Table 2). Moreover, foreign mothers’ children exhibited a higher risk of becoming overweight/obesity than Taiwanese children during the follow-up period (aOR: 1.51, 95% CI: 1.24–1.85). Other risk factors associated with overweight/obesity in children included age 2–7 years (aOR: 1.19, 95% CI: 1.08–1.31), male gender (aOR: 1.35, 95% CI: 1.23–1.47), and spectacle wear (aOR: 1.21, 95% CI: 1.01–1.44).

**Table 2:** Univariates and multivariate analyses of factors associated with overweight and obesity in children

Factors	Crude OR (95% CI)	P-value	aOR^a^ (95% CI)	P-value		
Low-income household	1.50 (1.35–1.66)	< 0.001	1.44 (1.29–1.60)	< 0.001		
Age, 2–7 yrs (vs. 0–1 yrs)	1.20 (1.09–1.32)	< 0.001	1.19 (1.08–1.31)	< 0.001		
Male gender	1.34 (1.23–1.47)	< 0.001	1.35 (1.23–1.47)	< 0.001		
Foreign mother	1.69 (1.39–2.05)	< 0.001	1.51 (1.24–1.85)	< 0.001		
Baseline dmft index^b^	1.01 (0.99–1.02)	0.317	1.00 (0.98–1.01)	0.736		
Spectacle wear	1.21 (1.01–1.44)	0.038	1.21 (1.01–1.44)	0.042		

aOR, adjusted odds ratio; CI, confident interval

^a^Adjusted for age, sex, mother’s nationality, baseline dmft index, spectacle wear

^b^dmft index: number of decayed teeth, missing due to caries and filled/restored due to dental caries for primary teeth

### Subgroup analysis for the association between household income and overweight/obesity in children

**Fig. 1:**
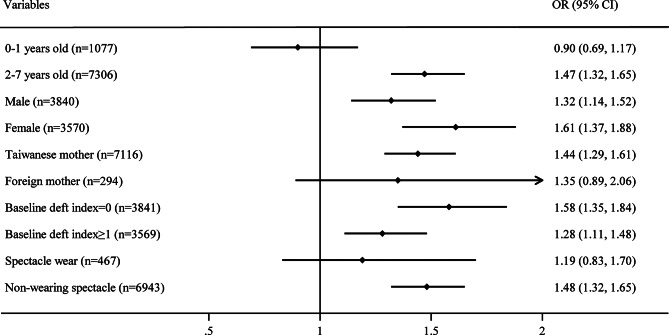
Subgroup analysis for the association between low-income household and overweight/obesity in Children Point estimate represents aOR. Each line represents 95% CI

Figure 1 shows the results of subgroup analysis of the association between low-income household and overweight/obesity after stratifying study participants by age, gender, mother’s nationality, baseline dmft index, and spectacle wear, respectively. Low-income households were associated with a higher risk of being overweight/obesity in children among all individuals’ subgroups except infants aged 0–1, foreign mothers’ children, and those wearing spectacles.

### The variations in BMI with age among children from low-income and non-low-income households

**Fig. 2:**
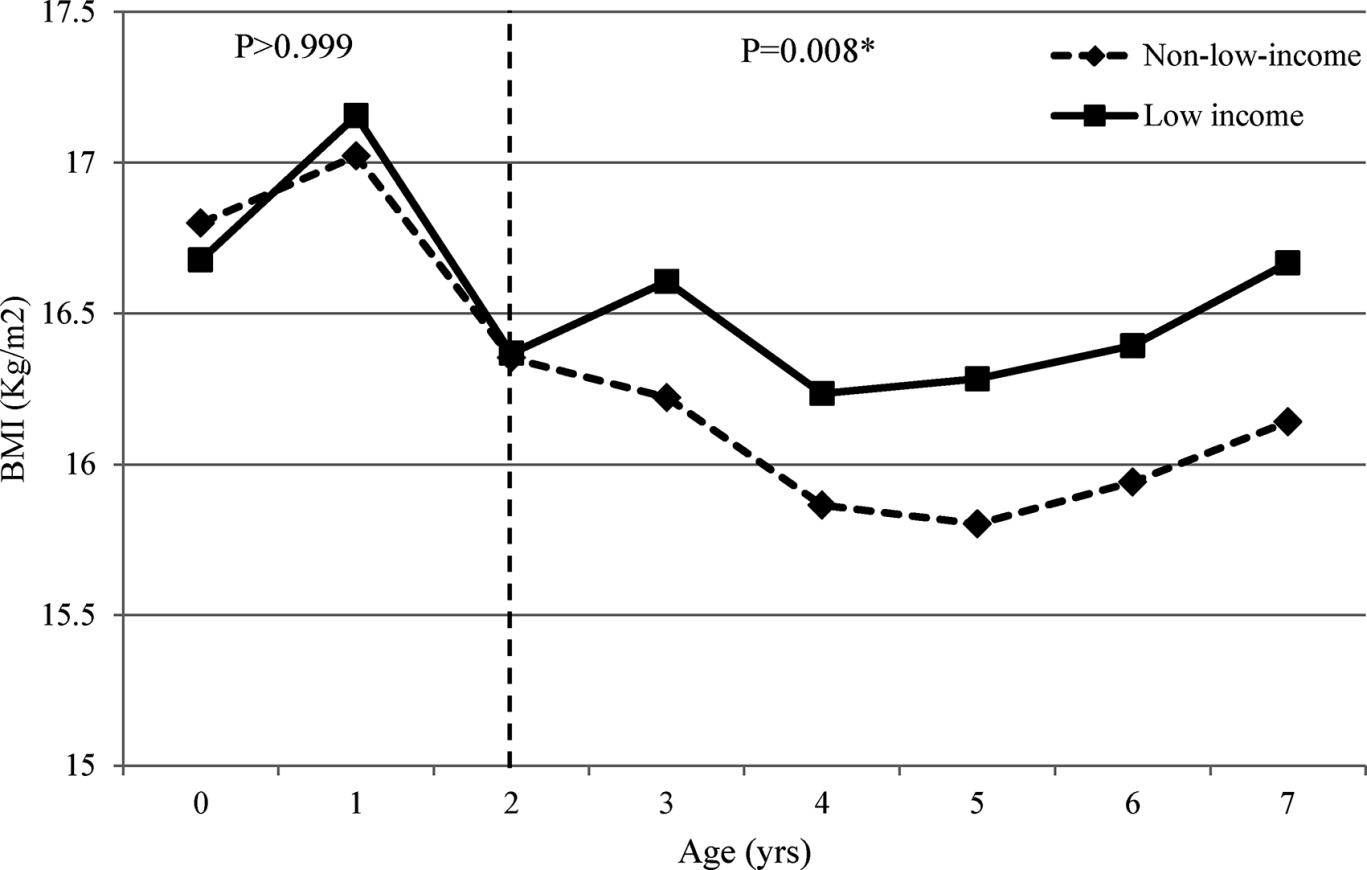
Variations in BMI with age in children from low-income households and non-low-income households. *Note* *Wilcoxon rank sum test

Figure 2 showed the variations in BMI with age among children from low-income and non-low-income households. Low-income household was significantly associated with a higher risk of becoming overweight/obesity in children aged 2–7 years (*P* =.01), but not significantly associated with a higher risk of becoming overweight/obesity in children aged 0–1 years (*P* >.999).

## Discussion

This prospective cohort study found that, compared to children from middle- and high-income household, those from low-income household had a significantly higher risk of becoming overweight/obesity during the follow-up period. Moreover, foreign mother’s children had a higher risk of becoming overweight/obesity than Taiwanese children during the follow-up period.

Our study showed robust association between belonging to low-income households and overweight/obesity, after stratifying study participants by age, gender, mother’s nationality, baseline dmft index, and spectacle wear, respectively. Low-income households were associated with a higher risk of becoming overweight/obesity in children among all individuals’ subgroups except infants aged 0–1, foreign mothers’ children, and those wearing spectacles.

Although several cross-sectional studies have reported that household income is reversely associated with children’s body weight [8, 9, 13], the temporality of household income level with overweight/obesity in children has not been extensively studied. Moreover, little study has been conducted to evaluate the impact of household income on the risk of childhood overweight/obesity over time. In our cohort study of 7,410 participants with repeated BMI measurements during a mean follow-up duration of 2.9 years, we found that preschool-aged children from low-income household were 1.4 times more likely to become overweight/obesity during the follow-up period from 0 to 7 years compared with children from middle- and high-income households. Our study findings suggest that future health promotion programs should target reducing overweight/obesity among preschool-aged children from low-income households.

Insufficiency family mealtimes, low affordability of healthy food, low levels of moderate to vigorous physical activity [27, 28], and inconsistent sleep schedules [29, 30] may explain for the higher risk of overweight/obesity in preschool-aged children living in a low-income family. A prior report showed that children in low-income households are less likely to regularly eat their main meals with their parents [3]. Moreover, lower income family was associated with a higher proportion of consuming foods with a low nutritional value [31–33]. As shared family mealtimes play an important role in promoting children’s healthy eating habits such as consuming more fruits and vegetables [3, 34], insufficiency family mealtimes could cause the unhealthy eating behaviors and subsequently increase the risk of overweight/obesity in children living in low-income families. Previous research has found a negative correlation, where less poverty was associated with more moderate to vigorous physical activity in children [27, 28, 35]. During times of increased economic stress, parents face additional challenges in managing the social, emotional, and economic stressors of daily life, and may have less time, energy, and resources available to provide positive and creative opportunities in parks, playgrounds, or even playtime at home [36]. This limitation in creating positive playtime experiences can contribute to childhood obesity. A longitudinal study found that children with inconsistent bedtime routines at the age of three faced a notably increased risk of obesity by the age of eleven [37]. Similarly, a study found that a consistent sleep schedule (delayed bedtime < 45 min on weekends) was a mediator between longer nighttime sleep duration and lower weight gain among preschoolers from low-income households [30].

Myopia in adolescents and children is also highly correlated with obesity. Myopia caused by obesity may be related to insulin resistance, one of the most common biochemical phenomena of obesity [38]. The most prevalent environmental factors contributing to myopia are associated with prolonged close eye use and insufficient time spent engaging in outdoor physical activities [39]. These factors are also correlated with BMI [40], and the risk of high myopia in obese children is 3.77 times [41]. The present study found that children who wear glasses have a higher risk of becoming overweight/obesity compared with children who do not wear glasses. In the subgroup analysis, the higher risk of becoming overweight/obesity among children who wear glasses only occurs among children from no-low-income households. If economic factors are considered, it may be possible that children from non-low-income households are more capable of purchasing mobile phones, tablets or game consoles, such that it is related to increasing the time spent using their eyes at close range and reducing the time engaged in outdoor physical activities.

The present study found that foreign mother’s children had a significantly higher risk of obesity than those of Taiwanese mothers. A prior report showed that transnational families in Taiwan have a relatively low SES, with foreign mothers usually being less educated and facing difficulties in lifestyle and language adaptation [42]. Our study findings suggest that future health programs to reduce the burden of overweight/obesity in children should particularly focus on the disadvantaged groups such as children of foreign mothers.

Taiwan currently provides various schooling, employment, medical and housing subsidies for low-income households [43]. Regarding nutritional supplement programs, the Taiwan government operates a lunch program in all public elementary school during the semester. Each school is responsible for choosing a healthy and affordable lunch package, and school kitchens or contracted restaurants prepare lunches accordingly. Poor children’s lunch fees are completely paid by the government [44]. However, Huang er al. (2015) highlighted that there may be a seasonal difference in the effectiveness of this lunch program [45]. Because it does not cover meals after school as well as meals during the holidays, it is recommended that schools, local governments and NGOs cooperate to provide complete nutritional supplement plans to make sure that needy children can continue to receive nutrition assistance in summer when school is not in session [45, 46]. It is also recommended that policymakers provide educational sessions and counseling services for parents on healthy eating for low-income families [47] or provide necessary nutrition education for children and teenagers, which will help low-income families establish healthy eating concepts [48].

The association between household income and childhood obesity in Taiwan is inconsistent [19–22]. Three studies have similar results to the present study [19–21]. Children from low-income families had a higher risk of becoming overweight/obesity compared with children from non-low-income families; however, Hsu et al. (2022) found a positive association between household income and BMI [22]. It may be caused by the bias in the income information collected by the questionnaire. To our knowledge, this cohort study is the first to determine the impact of household income on the risk of childhood overweight/obesity over time. Our study found that children from low-income household had a significantly higher risk of overweight/obesity. Nonetheless, some limitations should be considered when interpreting the findings of this cohort study. First, the data did not report breast-feeding prevalence and duration across income groups, and there is no available data on gestational diabetes and maternal obesity in this study. Considering that breast-feeding is regarded as an early life determinant of childhood obesity, it would indeed be interesting to explore these aspects in future research. Second, this study did not specifically provide information on parental education level, which is an important factor in understanding the socioeconomic context. Although we do not have a variable for parental education level, the important mediating factor related to obesity, lower affordability of healthy foods, is directly related to income. Indeed, Galobardes et al. (2006) highlighted that income is arguably the single best indicator of material living standards [49]. The results of our study also reflect the impact of income on childhood obesity. Third, our data currently extends only up to the year 2018. Future research endeavors could greatly benefit from the inclusion of a more extended observation period, allowing for a more robust exploration of the causal relationship between household income and obesity. Fourth, the generalizability of our results to other non-Asian ethnic groups requires further verification as all participants were Taiwanese.

## Conclusions

This prospective cohort study found that, compared to children from middle- and high-income households, those from low-income households had a significantly higher risk of being overweight/obesity during the follow-up period. The same result was found for foreign mother’s children compared to Taiwanese mothers’ children. Future health programs to reduce the burden of overweight/obesity in children should focus particularly on children from low-income households and children of foreign mothers.

## Data Availability

The datasets generated and/or analyzed during the current study are not publicly available due to the regulations of the information provider: Department of Health, Taipei City Government, but are available from the corresponding author on reasonable request.

## Abbreviations

aOR: adjusted odds ratio
BMI: body mass index
CI: confidence interval
dmft index: number of decayed teeth, missing due to caries and filled/restored due to dental caries for primary teeth
GEE: generalized estimating equations
OR: odds ratio
SD: standard deviation
SES: socioeconomic status
